# The Impact of Phenological Gaps on Leaf Characteristics and Foliage Dynamics of an Understory Dwarf Bamboo, *Sasa kurilensis*

**DOI:** 10.3390/plants13050719

**Published:** 2024-03-04

**Authors:** Chongyang Wu, Ryota Tanaka, Kyohei Fujiyoshi, Yasuaki Akaji, Muneto Hirobe, Naoko Miki, Juan Li, Keiji Sakamoto, Jian Gao

**Affiliations:** 1 Beijing for Bamboo & Rattan Science and Technology/International Centre for Bamboo and Rattan, Key Laboratory of National Forestry and Grassland Administration, Beijing 100102, China; wcy@icbr.ac.cn (C.W.);; 2 Faculty of Agriculture, Okayama University, Okayama 700-8530, Japan; 3 Biodiversity Division, National Institute for Environmental Studies, Tsukuba 305-8506, Japan; 4 Department of Environmental Ecology, Graduate School of Environmental and Life Science, Okayama University, Okayama 700-8530, Japan

**Keywords:** bamboo, sasa, beech forest, phenological gap, canopy, understory plant, plant morphology, plastically, leaf phenology

## Abstract

Phenological gaps exert a significant influence on the growth of dwarf bamboos. However, how dwarf bamboos respond to and exploit these phenological gaps remain enigmatic. The light environment, soil nutrients, leaf morphology, maximum photosynthetic rate, foliage dynamics, and branching characteristics of *Sasa kurilensis* were examined under the canopies of *Fagus crenata* and *Magnolia obovata*. The goal was to elucidate the adaptive responses of *S. kurilensis* to phenological gaps in the forest understory. The findings suggest that phenological gaps under an *M. obovata* canopy augment the available biomass of *S. kurilensis*, enhancing leaf area, leaf thickness, and carbon content per unit area. However, these gaps do not appreciably influence the maximum photosynthetic rate, total leaf number, leaf lifespan, branch number, and average branch length. These findings underscore the significant impact of annually recurring phenological gaps on various aspects of *S. kurilensis* growth, such as its aboveground biomass, leaf morphology, and leaf biochemical characteristics. It appears that leaf morphology is a pivotal trait in the response of *S. kurilensis* to phenological gaps. Given the potential ubiquity of the influence of phenological gaps on dwarf bamboos across most deciduous broadleaf forests, this canopy phenomenon should not be overlooked.

## 1. Introduction

Dwarf bamboos are widely found in the subalpine forests and certain subtropical mountain forests of Japan, South America, and Southwest China [1,2,3,4,5,6]. Dwarf bamboos typically occur as a dominant species covering forest floors with high density; as clonal plants, dwarf bamboos can share photosynthetic products between different culms through underground rhizomes, with culms spanning hundreds of square meters potentially belonging to the same genotype [7]. The shading effect and slowly decaying litter can inhibit the regeneration of some tree species, acting as ecological filters [8,9]. Dwarf bamboo forests also play other significant roles in forest ecosystems. For instance, more than 50% of the fine-root biomass in the surface soil layer was attributed to dwarf bamboos, which played a crucial part in the nutrient cycle [10]. Furthermore, when trees are felled, dwarf bamboos can mitigate nitrogen leaching from soil to rivers [11]. The interactions of dwarf bamboo with various organisms are also important factors affecting forest ecosystems. 

One of the most important causes of the dominance of dwarf bamboo communities in covering forest floors is their strong adaptability to changing light environments, whether under closed or open canopies with limited or abundant light intensity, respectively. The culm density, culm height, and biomass of *Sasa senanensis* were found higher in open sites than under closed canopies [12], while the net photosynthetic rate and leaf dry mass per unit area (LMA) were significantly lower in leaves under closed canopies than in canopy gaps [13]. The maximum photosynthetic rates of *Pleioblastus pubescens* were lower and the leaf lifespan was longer in shaded conditions than in open conditions [14,15]. The net photosynthetic rate and LMA were significantly lower in leaves under closed canopies than in canopy gaps in *Sasa kurilensis*, and the C/F ratio of ramets (the ratio of non-photosynthetic to photosynthetic parts) under a closed canopy was lower than in canopy gaps. The above research elucidates the plasticity of dwarf bamboo between high- and low-light environments from multiple perspectives.

Currently, research on the physiological and ecological characteristics of dwarf bamboos has mainly focused on differences in low- and high-light environments. However, a phenological phenomenon exists in deciduous broad-leaved forests that has not yet received widespread attention. In these forests, the canopy of *Fagus crenata* completes leaf-emergence earlier in early spring and leaf-fall later in autumn than that of *Magnolia obovata*. This results in an increase in temporal light availability under the *M. obovata* canopy during these periods. [16,17,18,19]. Tomita and Seiwa (2004) have shown that *S. kurilensis* and *Sasa palmata* show a higher Sasa quantity index under *M. obovata* canopies than under *F. crenata* canopies [18]. The culm density and biomass of *S. kurilensis* are greater under *M. obovata* and *Betula grossa* canopies than under *F. crenata* canopies [16]. Only a few studies have indicated the impact of the phenological gap on the community structure of dwarf bamboo growing on the forest floor.

The ability of plants to adapt to heterogeneous light environments is crucial for their survival and reproduction. This adaptation is achieved primarily through adjustments in leaf morphology [20,21,22,23], photosynthetic performance [23,24], and changes in leaf chemical composition [21,24]. There have been no investigations into the adaptations of leaf characteristics to such temporally heterogeneous light environments in mixed-species canopies. The objectives of this study are as follows: (1) to confirm whether phenological gaps affect the leaf characteristics, leaf dynamics, and branching characteristics of *S. kurilensis*; (2) to investigate whether these effects vary with the culm age of *S. kurilensis*; and (3) to discuss the plasticity of *S. kurilensis* leaves and branches in response to phenological gaps and understand their survival strategies under the forest canopy.

## 2. Results

### 2.1. Phenological Gaps and Microhabitat Conditions under Different Canopy Species

**Figure 1:**
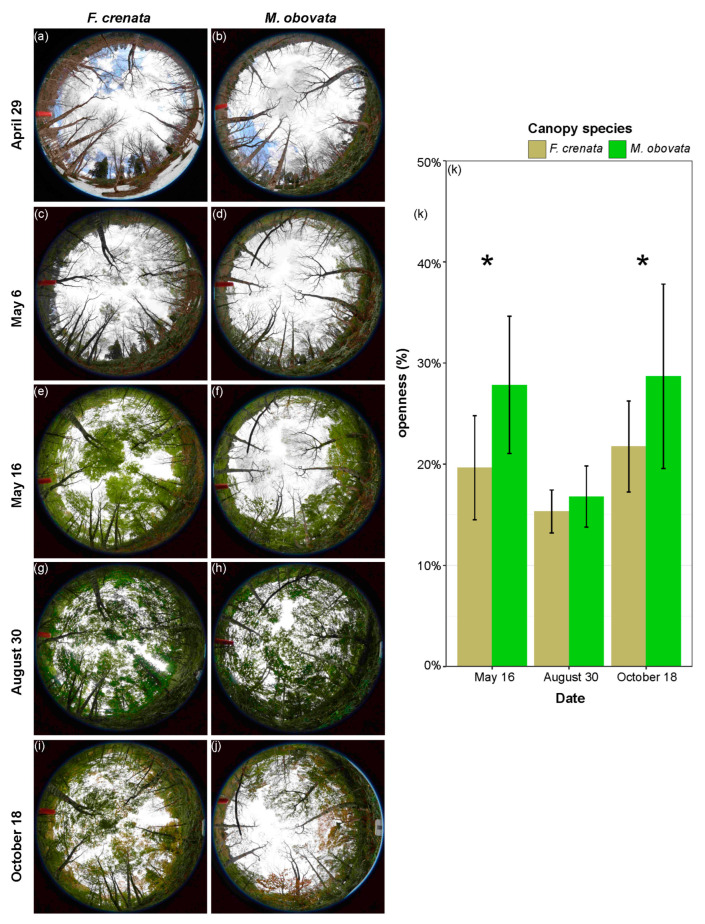
Hemispherical photos showing phenological gaps and canopy openness under two canopy species: left, *Fagus crenata*; and right, *Magnolia obovata*. Photos acquired on (**a**,**b**) 29 April; (**c**,**d**) 6 May; (**e**,**f**) 16 May; (**g**,**h**) 30 August; and (**i**,**j**) 18 October. (**k**) Graph of percent canopy openness on 16 May, 30 August, and 18 October for *F. crenata* and *M. obovata*. The asterisk indicates a significant difference in openness between the two types of canopy trees. Error bars represent standard deviation. The detailed results of the Bayesian models are shown in Supplementary Table S1.

Figure 1a–j shows the process of how phenological gaps occur from April to October using hemispherical photos. Both *F. crenata* and *M. obovata* canopies were still leafless on 24 April, while the ground was still covered with snow. By 6 May, the *F. crenata* canopy had started to leaf out, while the *M. obovata* canopy remained leafless. On 16 May, the *F. crenata* canopy had fully leafed out, while the *M. obovata* canopy was just beginning to leaf out. We believe that at this time, a phenological gap had formed beneath the *M. obovata* canopy, with significantly higher values of canopy openness compared to those beneath the *F. crenata* canopy (Figure 1k; Supplementary Table S1). By 30 August, both the *F. crenata* and *M. obovata* canopies had closed, with no significant differences in canopy openness beneath the canopies. Another phenological gap occurred around 18 October, as the *M. obovata* canopy had more leaf-drop than the *F. crenata* canopy, resulting in a significant difference in canopy openness under the trees of these two species (Figure 1k; Supplementary Table S1).

In addition to the light environment, the slope, convexity, soil water content, soil carbon content, and soil nitrogen content of all grids were compared to confirm whether there were other micro-environmental differences between the two types of canopies surveyed here (Table 1, Supplementary Table S2). No significant differences were observed in these other micro-environments. To minimize the impact of other unknown factors between different sub-plots on the dependent variable, a number of sub-plots were treated as random effects in our mixed-effects models. This approach allowed group-level differences to be accounted for while reducing the influence of these unknown factors on the outcome variable.

### 2.2. Leaf Characteristics under Different Canopy Species

**Figure 2:**
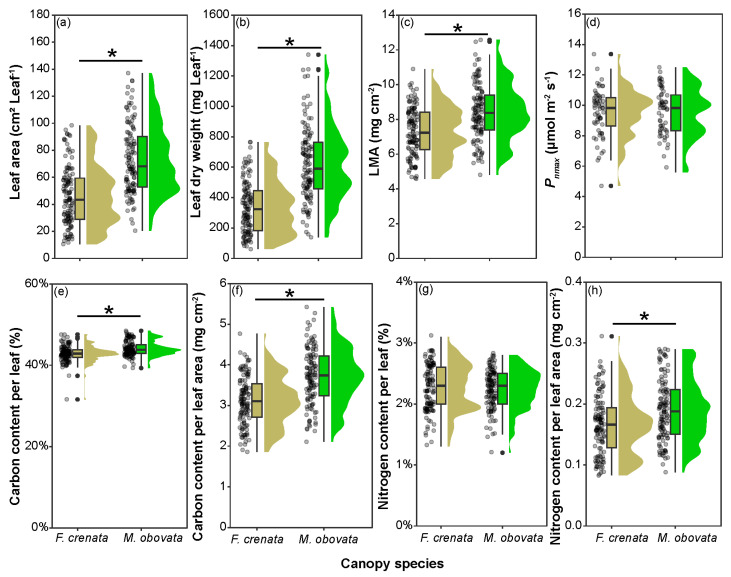
Comparison of *Fagus crenata* and *Magnolia obovata* leaf characteristics: (**a**) leaf area; (**b**) leaf dry weight; (**c**) leaf dry mass per unit area (LMA); (**d**) maximum leaf photosynthetic rate (*P_nmax_*); (**e**) carbon content per leaf; (**f**) carbon content per leaf area; (**g**) nitrogen content per leaf; and (**h**) nitrogen content per leaf area of *Sasa kurilensis* under two canopy species. Points represent measured values, colored blocks represent the data distribution, and * represents a significant difference between these two canopy trees. The detailed results of the Bayesian models are shown in Supplementary Table S3.

**Figure 3:**
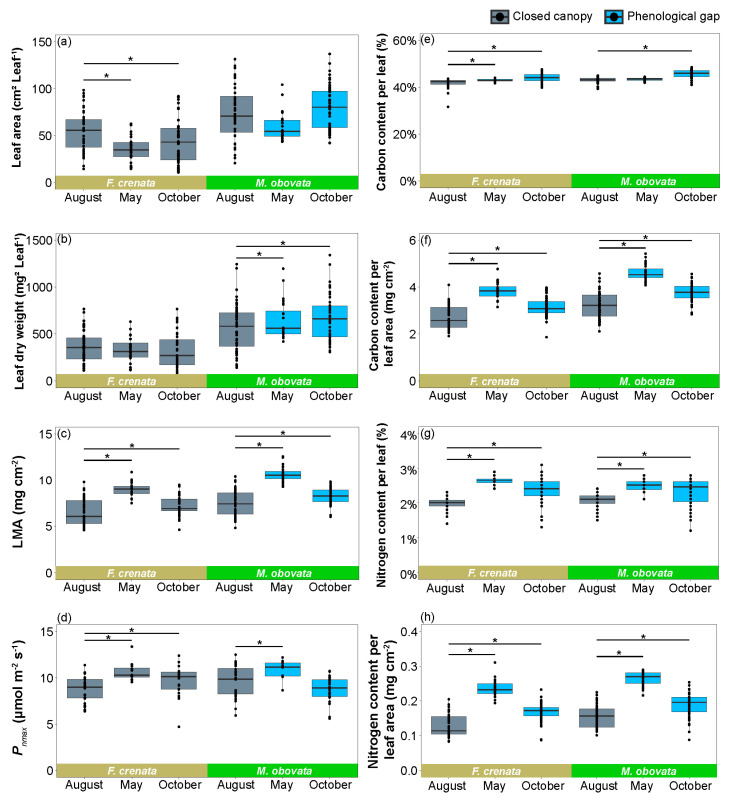
Box plots of the characteristics of *Sasa kurilensis* in May, August, and October under canopy trees of *Fagus crenata* and *Magnolia obovata*: (**a**) leaf area, (**b**) leaf dry weight, (**c**) leaf dry mass per unit area (LMA), (**d**) maximum leaf photosynthetic rate (*P_nmax_*), (**e**) carbon content per leaf, (**f**) carbon content per leaf area, (**g**) nitrogen content per leaf, and (**h**) nitrogen content per leaf area. Grey and blue shading indicate closed canopies and phenological gaps, respectively. * represents a significant difference in August or October when compared with May. The detailed results of the Bayesian models are shown in Supplementary Table S4.

We compared the leaf area, leaf dry weight, LMA, maximum net photosynthetic rate (*P_nmax_*), carbon content per leaf, carbon content per leaf area, nitrogen content per leaf, and nitrogen content per leaf area of *S. kurilensis* under each of the two canopy species. Except for *P_nmax_* and nitrogen content per leaf, *S. kurilensis* grown under an *M. obovata* canopy had significantly higher values than those under an *F. crenata* canopy (Figure 2; Supplementary Table S3). We then analyzed whether differences had been observed in these leaf characteristics during different seasons based on when the phenological gaps occurred and when canopies were closed (Figure 3; Supplementary Table S4). The results showed that compared to August, LMA, carbon content per leaf area, nitrogen content per leaf, and nitrogen content per leaf area of leaves in May and October had increased significantly under both *F. crenata* and *M. obovata* canopies. However, the leaf area in May and October significantly decreased under the *F. crenata* canopy but not under the *M. obovata* canopy. The leaf dry weight increased significantly in May and October under the *M. obovata* canopy, but not under the *F. crenata* canopy. Similarly, *P_nmax_* and carbon content per leaf also showed significant increases in May and October under an *F. crenata* canopy, but with different tendencies observed under an *M. obovata* canopy. 

### 2.3. The Influence of Phenological Gaps on Leaf Characteristics

**Figure 4:**
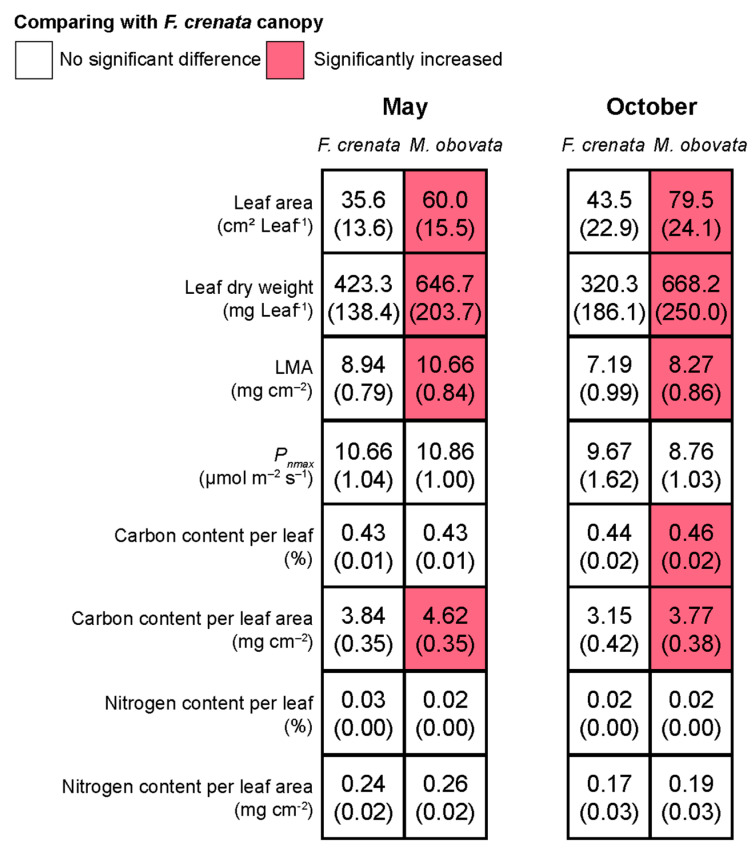
The effects of phenological gap on the leaf characteristics of *Sasa kurilensis* in May and October. Numbers represent mean values, and the numbers in parentheses indicate the standard deviation; pink shading indicates significant differences between canopy trees of *Fagus crenata* and *Magnolia obovata*. The detailed results of the Bayesian models are shown in Supplementary Table S5.

Although various leaf characteristics such as leaf area, leaf dry weight, LMA, and others exhibited significant differences in different seasons, it was unclear whether these differences were a result of intrinsic phenological changes in *S. kurilensis* or the influence of phenological gaps. Therefore, the differences in leaf characteristics were further compared between the two canopy species in May and October when phenological gaps appeared (Figure 4; Supplementary Table S5). The results showed that in May and October, leaf area, leaf dry weight, LMA, and carbon content per leaf area under an *M. obovata* canopy were significantly higher than those under an *F. crenata* canopy. In October, the carbon content per leaf under an *M. obovata* canopy was also significantly higher than that under an *F. crenata*. This indicates that these leaf characteristics of *S. kurilensis* are influenced by the presence of phenological gaps.

**Figure 5:**
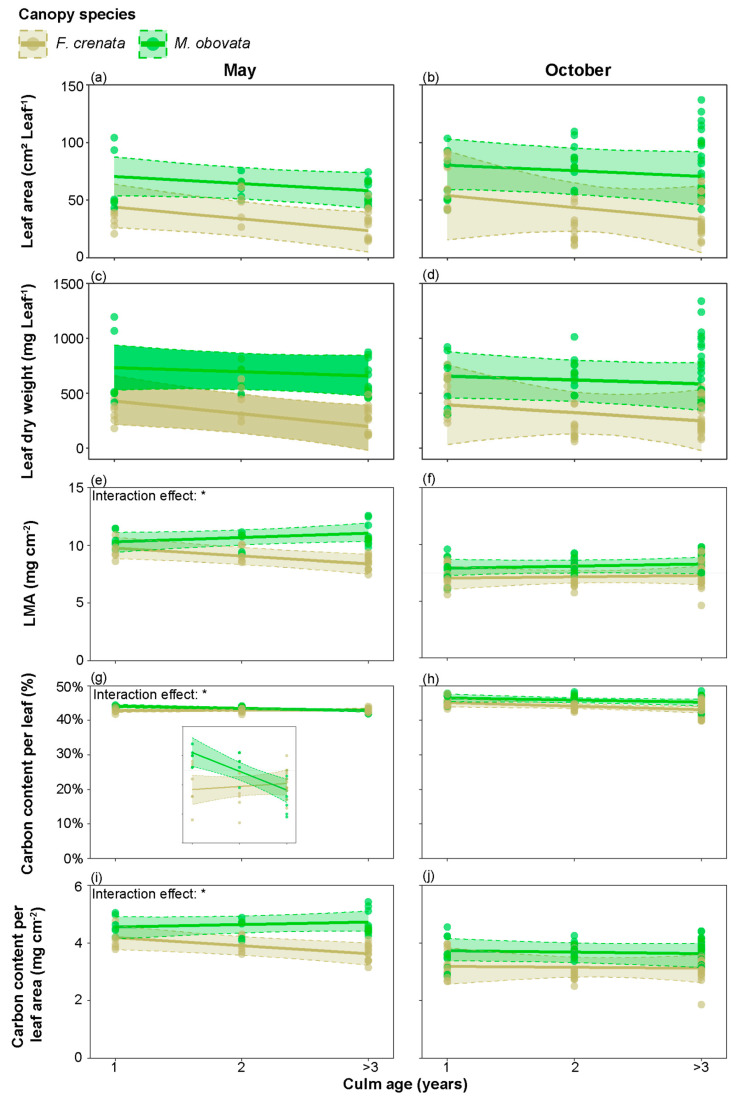
The interaction effects between canopy species and culm age on: (**a**,**b**) leaf area; (**c**,**d**) leaf dry weight; (**e**,**f**) leaf dry mass per unit area (LMA); (**g**,**h**) carbon content per leaf; and (**i**,**j**) carbon content per leaf area of *Sasa kurilensis* in (left) May and (right) October. Points represent measured values; lines represent predictions using the mean of the posterior distribution; shaded blocks represent the 95% Bayesian credibility intervals; * represents a significant difference in interaction effects between canopy trees of *Fagus crenata* and *Magnolia obovata*. An inset is provided to better visualize the subtle differences among the data points in g. The detailed results of the Bayesian models are shown in Supplementary Table S6.

Figure 5 shows the interaction effects of canopy species and the culm age of *S. kurilensis* on five leaf characteristics in May and October (Supplementary Table S6). The results were used to assess whether different culm ages affected the promotion of leaves in phenological gaps. The results showed that in May, the promotion effects on LMA, carbon content per leaf, and carbon content per leaf area under *M. obovata* were influenced by culm age. Specifically, LMA and carbon content per leaf area increased significantly with culm age, while carbon content per leaf decreased significantly. However, in October, no similar trends were observed in these leaf characteristics.

### 2.4. Foliage Dynamics, Branching Characteristics and Above Ground Biomass under Different Canopy Species

**Figure 6:**
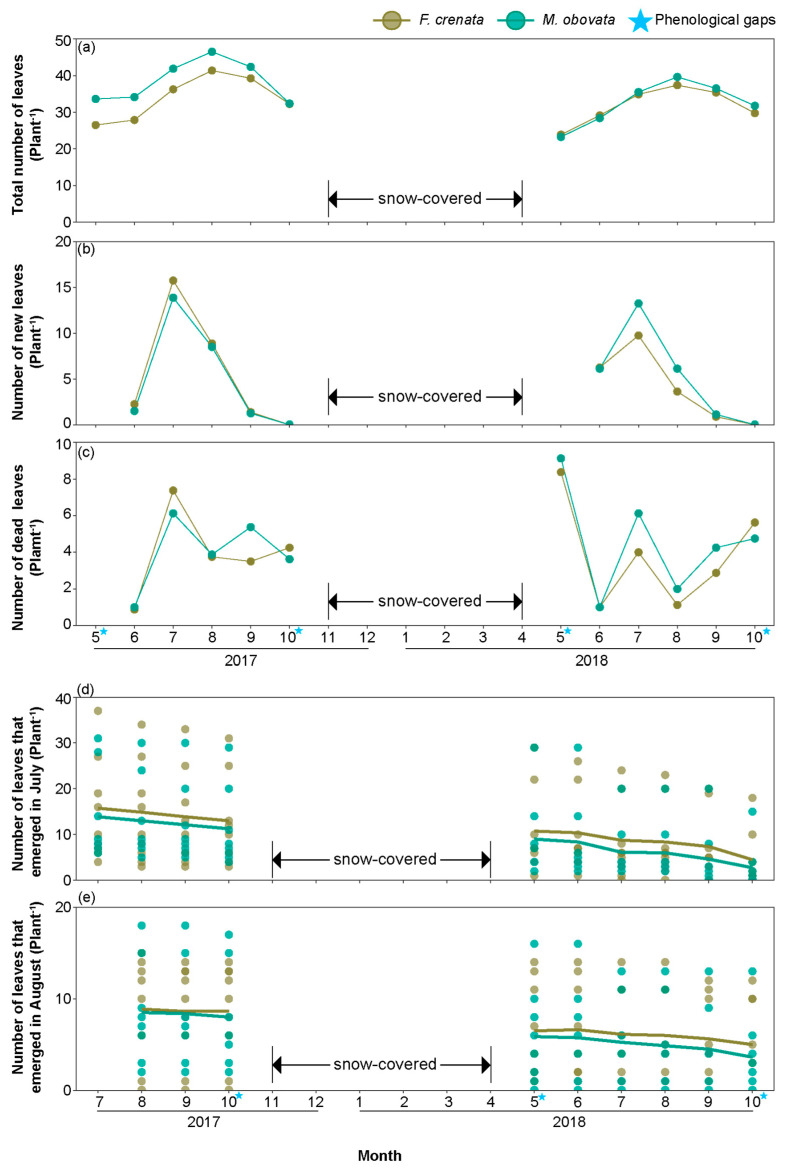
Monthly leaf characteristics of *Sasa kurilensis* under the canopy trees of *Fagus crenata* and *Magnolia obovata* from 2017 to 2018: (**a**) total number of leaves; (**b**) number of new leaves; (**c**) number of dead leaves; (**d**) number of leaves that emerged in July; and (**e**) number of leaves that emerged in August. The *S. kurilensis* in the study area is entirely covered by snow from October to May annually; ★ represents the months with a phenological gap; points represent mean values in (**a**–**c**); and points and lines represent measured values and mean values in (**d**,**e**). The detailed results of the Bayesian models are shown in Supplementary Table S7.

**Figure 7:**
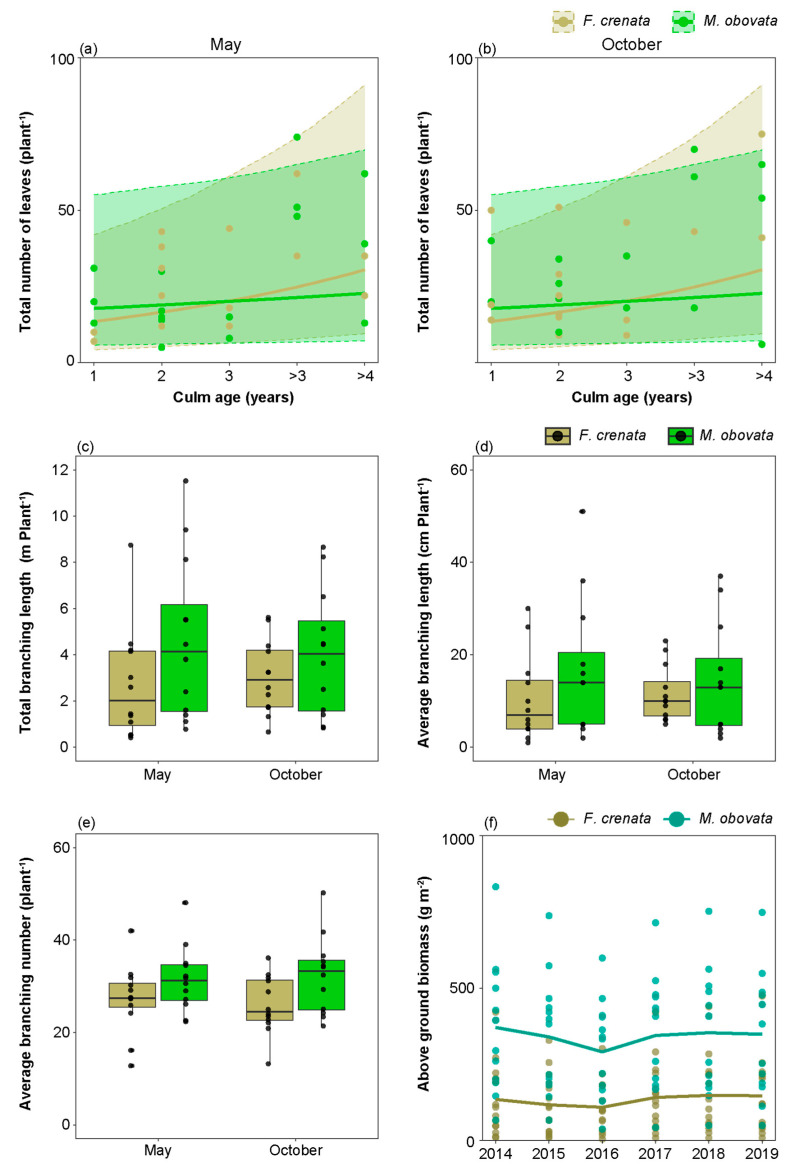
Comparative influence of two canopy species on *Sasa kurilensis*. (**a**,**b**) The interaction effects with culm age of *Sasa kurilensis* in May and October; points, lines, and shaded blocks represent measured values, predictions using the mean of the posterior distribution, and 95% Bayesian credibility intervals, respectively. (**c**–**e**) Branching characteristics of *S. kurilensis* under both canopy trees in May and October: total branch length (**c**), average branch length (**d**), and average number of branches (**e**). (**f**) Interannual variation of the above-ground biomass of *S. kurilensis* under the two canopy tree species from 2014 to 2019; points represent measured values of each sub-plot, and lines represent mean values. The detailed results of the Bayesian models are shown in Supplementary Table S8–S10.

Figure 6a–c depicts the monthly variations in the total number of leaves, new leaves, and dead leaves of *S. kurilensis* under the two canopy species analyzed here over two years (Supplementary Table S7). No significant differences were observed in the total number of leaves, new leaves, and dead leaves among different canopy types in all months. However, compared to an *M. obovata* canopy, the total number of leaves under an *F. crenata* canopy significantly increased with culm age in May and October (Figure 7a,b; Supplementary Table S8). A greater number of leaves of *S. kurilensis* emerged in July and August than in other months, and we visualized the changes in the quantities of the leaves that emerged in July and August over two years (Figure 6d,e; Supplementary Table S7). It can be seen that the mortality curves of leaves of the same age were almost identical under different canopies, indicating that almost no difference in leaf lifespan existed between the *F. crenata* and *M. obovata* canopies.

The analysis of whether phenological gaps had an effect on the branching characteristics of *S. kurilensis* found no significant differences in the total branching length, average branching length, and average branching number under the two canopy species in both May and October (Figure 7c–e; Supplementary Table S9).

We estimated the above ground biomass of *S. kurilensis* in 28 sub-plots from 2014 to 2019 based on the ground diameter. The results showed that under both *F. crenata* and *M. obovata* canopies, no significant increasing or decreasing trend in the above ground biomass of *S. kurilensis* was observed over the six year period, indicating that the *S. kurilensis* community remained stable under canopies of both species (Figure 7f; Supplementary Table S10).

## 3. Discussion

### 3.1. Seasonal Variations in the Leaf Characteristics of S. kurilensis

Our observations and a previous study [18] indicated that the temporal persistence of phenological gaps lasts for approximately 10–20 days (Figure 1). Due to the variation in leaf phenology among many tree species [25], phenological gaps may be widespread in forests. *Sasa kurilensis* under two canopy species exhibited significant seasonal variations in leaf characteristics (Figure 3); an interesting point is that the seasonal changes in leaf area and leaf dry weight of *S. kurilensis* are completely different under *F. crenata* and *M. obocata* canopies. Compared to August, the LMA of *S. kurilensis* significantly increased in May and October under both the canopies. Concurrently, the leaf area of *S. kurilensis* significantly decreased under the *F. crenata* canopy, while the leaf dry weight of *S. kurilensis* significantly increased under the *M. obovata* canopy. Based on previous studies and the results of the present study, it appears that the available light resources and biomass for *S. kurilensis* under an *F. crenata* canopy must be less than those under an *M. obovata* canopy (Figure 1) [16,18]. *Sasa kurilensis* growing under an *M. obovata* canopy can enhance the LMA by increasing the allocation of biomass per unit leaf area, whereas *S. kurilensis* under an *F. crenata* canopy is constrained by allocatable resources and can only achieve the same result through a reduction in leaf area. *Sasa kurilensis*, which is covered by snow and experience more than five months of darkness, is highly likely to suffer from photoinhibition or other types of damage when it is suddenly exposed to the year’s strongest light environment during snowmelt [26]. The significant increase in LMA results in thicker leaves, which not only effectively mitigates the effects of photoinhibition [27] but also enhances carbon and nitrogen allocation per leaf area, thereby promoting improved photosynthetic performance [28]. A significant increase in LMA, *P_nmax_*, and carbon and nitrogen allocation per leaf and per unit leaf area of *S. kurilensis* were observed in May and October; this is a reasonable adaptation to their environment, regardless of whether under the canopy species of *F. crenata* or *M. obovata*.

### 3.2. The Impact of the Phenological Gap on the Leaf Characteristics of S. kurilensise

The effect of phenological gaps on leaf characteristics is concealed within the inherent seasonal variations of *S. kurilensis*. During May and October, the phenological gaps increase leaf area, leaf dry weight, LMA, and carbon content per leaf area significantly under an *M. obovata* canopy (Figure 4); this should be due to the differences in available light resources. We believe that the *S. kurilensis* under an *M. obovata* canopy, which benefits from phenological gaps, also aids the *S. kurilensis* under an *F. crenata* canopy through physiological integration. We make this inference because in pure forests of *F. crenata*, the LMA in *S. kurilensis* is 5.32 ± 0.20 (mg cm^−2^), which is much lower than the results (7.30 ± 1.40 mg cm^−2^) of this survey (Figure 2); this implies that in pure forests without phenological gaps, the leaves of *S. kurilensis* can become thinner [17]. The creation of phenological gaps improves the light environment, which in turn promotes the dominance of *S. kurilensis* across the forest floor. This dominance is contingent upon the plasticity of their leaves and their strong ability for physiological integration. In some plant communities, the age structure of the population and the functional roles of individuals can vary under different light environments [29]. Based on this, we considered whether the plasticity of leaves in response to phenological gaps varies with culm age. The results showed that as culm age increased, both leaf area and leaf dry weight decreased almost in parallel in both canopies. Under the *F. crenata* canopy, the LMA and carbon content per leaf significantly decreased with increasing culm age (Figure 5). For the light-limited environment under an *F. crenata* canopy, reducing material allocation to older culms to ensure larger leaf area and higher LMA in younger culms for photosynthesis production may be a cost-effective strategy. This capability allows for adaptation to diverse light environments, and studies have shown that *S. kurilensis* invades subalpine and alpine ecosystems through its high stress tolerance and adaptability to climate change, where significant differences exist in the growth rates of the current year and previous year culms across forest, subalpine, and alpine ecosystems. [30].

### 3.3. The Impact of Phenological Gaps on the Foliage Dynamics and Branching Characteristics of S. kurilensis

In this study, no significant difference was observed in the total number of leaves and number of new and dead leaves per culm under both canopy species over a two year period (Figure 6a–c). However, considering that the leaves under an *M. obovata* canopy were larger and thicker, the foliage biomass per plant under an *M. obovata* canopy was higher than that under an *F. crenata* canopy, which is consistent with earlier studies. Tobita et al. (2006) reported that dwarf bamboo grown in high-light environments has greater leaf biomass per unit area (g m^−2^) than those grown in low-light environments [13].

This trend has also been observed in other plant species [31]. *Sasa kurilensis* under the canopy of *M. obovata* invested in larger and thicker leaves to increase photosynthetic performance, rather than increasing the number of leaves. *Sasa kurilensis* does not produce new leaves repeatedly on the same branch, and every year leaves emerge on new branches. Although we did not make precise calculations, it is evident that this approach requires the plants to expend more energy and resources. Some components in leaves, such as nitrogen, can even be recycled with an individual plant according to demand [32], but it is unlikely that the material allocated to new branches can be recycled. Our analysis of branching characteristics also confirms this inference, because no difference was observed in total and average branch length alone with the number of branches between *S. kurilensis* under both the canopy types analyzed here (Figure 7c–e). Although the *M. obovata* canopy has phenological gaps that can temporarily result in an increase in available light resources, most of the time, *M. obovata* still has a closed canopy, as does *F. crenata*. Even if increasing branch length places leaves in more extended positions, little may be gained in the production of photosynthetic products during the closed-canopy period. Additionally, more branches may increase the risk of intraspecific competition and self-shading [33,34,35,36,37].

In general, thicker leaves are associated with longer lifespans [38] because the investment in leaf tissue needs to be recovered before the leaf dies. The results of the present study showed that the leaf lifespans of *S. kurilensis* under both canopy species were almost identical (Figure 6), and estimated to be around 2–3 years. *Sasa kurilensis* under an *F. crenata* canopy allocated fewer resources to leaves but achieved the same leaf lifespan as *S. kurilensis* under an *M. obovata* canopy. We considered two reasons for this: first, the return on investment under an *F. crenata* canopy was higher; second, physiological integration provided resource compensation, extending the lifespan of leaves under an *F. crenata* canopy. Evidence to support this has been found in other studies; for example, culm lifespan was significantly longer under low- than under high-light conditions [6]. The advantage of this strategy was that it allowed for the long-term recovery of the initial investment when the culm lifespan was long. During the years the present investigation was conducted, no signs of decline were observed in the *S. kurilensis* community under both canopy species analyzed here (Figure 7f). *Sasa kurilensis* under the *M. obovata* canopy expands its leaf area to adapt to the transient appearance of phenological gaps, thereby gaining more photosynthetic products. Our results also imply that these photosynthetic products might be shared with other culms experiencing a greater scarcity of light resources through physiological integration. While we cannot confirm this hypothesis at present, we believe that the strong plasticity of *S. kurilensis* leaves is one of the key reasons for its ability to occupy the forest floor.

## 4. Materials and Methods

### 4.1. Study Area

**Figure 8:**
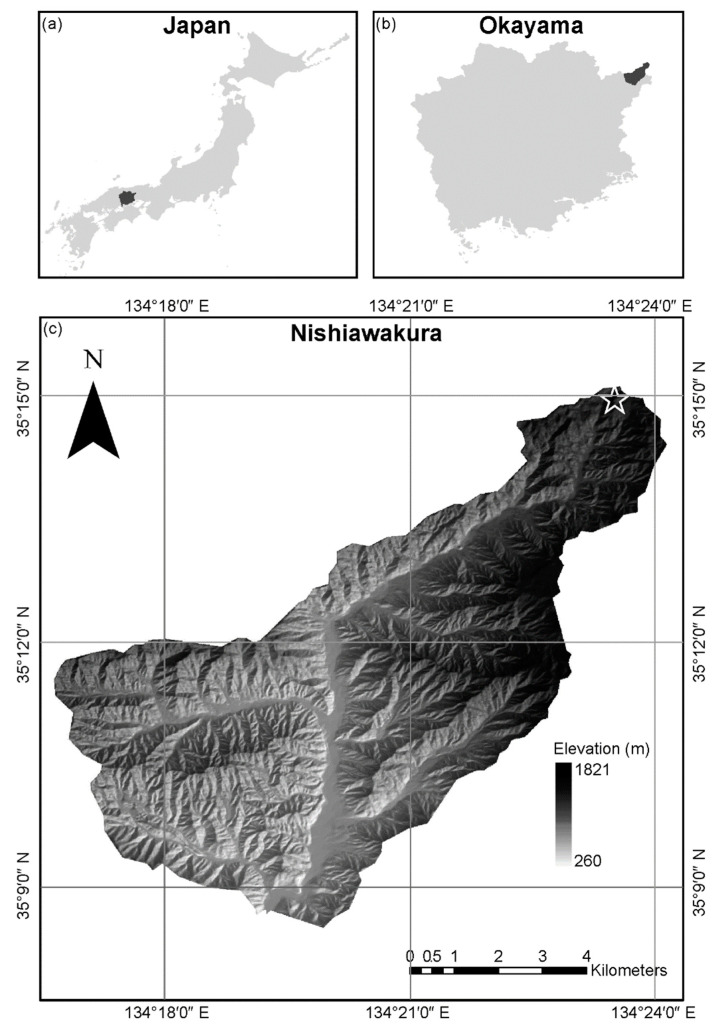
Map of the study area: (**a**) Okayama Prefecture in Japan; (**b**) Nishiawakura village within Okayama; and (**c**) a topographical map of Nishiawakura village. The stars indicate the locations of the study plot.

The study was conducted in an old-growth beech forest located in Wakasugi Forest Reserve, at 35°14′ N, 134°23′ E, at 1048 m above sea level, in the Chugoku Mountains near the village of Nishiwakura, in Okayama Prefecture, in western Japan (Figure 8). The soil is a brown forest soil on granite rock substrate. The site falls within the cool temperate zone, with a mean temperature of approximately 8.2 °C and an average annual precipitation of 2400 mm. In winter, *S. kurilensis* is completely covered by accumulated snow. The canopy layer of the forest is dominated by *F. crenata*, *M. obovata*, and *B. grossa* [39]. The forest floor is densely covered with *S. kurilensis*, with *F. crenata*, *Acer japonicum*, *Acer mono*, and *Viburnum furcatum* as the dominant species in the canopy layer.

### 4.2. Establishment of Study Plot

A rectangular 10 m × 120 m survey plot was established, which was subdivided into 48 sub-plots, each measuring 5 m × 5 m; in addition, a 2 m × 2 m sub-plot was established at the center of each larger 5 m × 5 m sub-plot. The assessments of the light environment and soil nutrients were conducted in the 5 m × 5 m sub-plots, while all other investigations were performed within the 2 m × 2 m sub-plots (Supplementary Figure S1).

### 4.3. Leaf Characteristics 

In August 2017, October 2017, and May 2018, leaves that were not shaded, from culms aged one, two, and over three years, were randomly sampled in the sub-plots (Supplementary Figure 1). Throughout the study, a total of 238 leaves from 65 culms with different ages were randomly collected and taken back to the laboratory for measurements. The leaves were scanned and their area was determined using imaging software (LIA for Win 32 version 0. 3781 by K. Yamamoto, Nagoya Univ., Nagoya, Japan). Subsequently, the leaves were dried at 70 °C for more than 48 h using an oven, and their dry weight was measured. The LMA, leaf area, and dry weight per leaf were then calculated for each individual leaf. Carbon and nitrogen content in each leaf were determined using an elemental analyzer (2400 II CHNS/O, Perkin-Elmer, Norwalk, CT, USA).

### 4.4. Leaf Maximum Net Photosynthetic Rate 

The experiments on the *P_nmax_* of non-shaded *S. kurilensis* leaves were taken during the appearance of phenological gaps in May and October and during closed-canopy conditions in August in both 2016 and 2017 (Supplementary Figure S1). To ensure consistency, measurements for each season were completed within a consecutive five day period. The measurements were conducted on sunny days between 9:00 and 11:30 AM, ensuring that the leaves had been unshaded for over an hour under natural conditions before sampling. Prior to measurements, leaves were exposed to a light intensity of 1800 μmol m^−2^ s^−1^ for half an hour using an open gas exchange system (LI-6400, LI-COR, Lincoln, NE, USA) to fully open the stomata before measuring maximum photosynthesis rates (this intensity was confirmed in a preliminary experiment to not cause photoinhibition in *S. kurilensis* leaves). A total of 120 non-shaded leaves were measured under *F. crenata* and *M. obovata* canopies. The *P_nmax_* was measured under the following conditions: a photosynthetic photon flux density of 1600 μmol m^−2^ s^−1^, a CO_2_ concentration of 370 parts per million, and a relative humidity of 65–75%. The leaf temperature was set to be the same as the air temperature. 

### 4.5. Foliage Dynamics and Branching Characteristics

A two year survey (2017–2018) was conducted to investigate the foliage dynamics of *S. kurilensis* under two canopy species listed above (Supplementary Figure S1). For each species, we randomly selected eight non-shaded culms of different ages (one, two, three, and over four years). The study site, where *S. kurilensis* grows, experiences snow cover from December to April every year. The total number of leaves on each culm was recorded every month from May to October, while dead and new leaves were accurately tracked by marking them with different colored markers. In addition, we investigated the branching characteristics of 12 randomly selected culms under each canopy species, recording the number and length of all branches during both phenological gaps (May and October) and closed-canopy (August) conditions in 2018.

### 4.6. Estimation of Above Ground Biomass

We marked all individuals of *S. kurilensis* growing in each sub-plot and carried out a culm tracking survey every October from 2014 to 2019 (Supplementary Figure S1). The survey included ground diameter and the numbers of surviving, dead, and new culms. The above ground biomass of *S. kurilensis* in each of the 28 smaller sub-plots was estimated based on ground diameter measurements. The data of above ground biomass for the years 2014 and 2015, as well as the calculation method for biomass (Equation (1)), can be found in Wu et al. [16].

Log(*W*) = 1.49 × Log(*D*^2^) + 1.78 (*r* = 0.92, *p* < 0.001) (1)

*W*: above ground biomass *D*: ground diameter.

### 4.7. Light Environment and Microhabitat Conditions 

Canopy openness was measured at a height of 3 m at the center of each 28 sub-plot (Supplementary Figure S1). Hemispherical photographs were taken on 29 April, 6 May, 16 May, 30 August, and 18 October 2017 to document when phenological gaps occurred or disappeared. Canopy openness was calculated using LIA32 analysis software.

An investigation into the soil nutrient conditions of each grid was conducted in October 2018 (Supplementary Figure S1). Soil samples were collected from the four corners and the center of each 5 m × 5 m sub-plot, the A0 layer was removed, and approximately 50 g of hard soil was collected from a depth up to 50 cm; each set of five samples from one sub-plot were subsequently combined prior to analysis. Stones and plant roots were removed using a 2 mm sieve and the soil was weighed in its wet state. After drying for one week in a well-ventilated room, the soil was placed in an oven and dried at 70 °C for 48 h. The dry weight of soil sample was measured and the moisture content of each sample was calculated. The carbon and nitrogen content of soils were measured using a CN coder (JM1000CN, J-Science Laboratory, Kyoto, Japan). Slope and convexity in each of the 28 grids were derived from Akaji et al. [40].

### 4.8. Model Analysis

To investigate the impact of phenological gaps on the individual leaves, foliage, and branching characteristics of *S. kurilensis*, a generalized linear mixed model (GLMM) was constructed using the Bayesian Markov Chain Monte Carlo method. The use of Bayesian modelling methods present various benefits, such as allowing for distribution-type selection of the data, and providing effective data fit even with limited sample numbers. Hence, Bayesian analyses are widely utilized across numerous fields of study [41,42,43]. The dependent variables included leaf area (cm^2^ leaf^−1^), leaf dry weight (mg leaf^−1^), LMA (mg cm^−2^), *P_nmax_* (μmol m^−2^ s^−1^), carbon and nitrogen content per leaf (%), carbon and nitrogen content per leaf area (mg cm^−2^), total number of leaves (plant^−1^), number of dead and new leaves (plant^−1^), total length of branches (m plant^−1^), average length of branches (cm), average number of branches (plant−1), and above ground biomass (g m^−2^). The independent variables were canopy species (*F. crenata* and *M. obovata*), season (May, August, and October), and culm age (one, two, three, over three, and over four years). For analyses of the photosynthetic performance of leaves, the random effect was set to individual *S. kurilensis* identifiers; for other analyses, the random effect was set to sub-plot identifiers. Supplementary Table S11 contains detailed information on all model structures. Topography of each sub-plot was set as a random effect when significant differences were observed between two canopy species. In the absence of such significant differences, the number of sub-plots was set as a random effect (Supplementary Table S12 and S13). For each model, we ran four independent chains with one interval, and obtained 5000 posterior samples from each chain after a burn-in of 5000 iterations using non-informative prior distributions since we did not have any prior information for our data. We considered a 95% credible interval that did not include zero to be statistically significant for the mean of the variables. We calculated the R-hat of all parameters and ensured that it was less than 1.1. All models were performed using the brms package [44] on R version 4.2.2 and R Studio version 1.3.1093.

## Figures and Tables

**Table 1:** Differences in topography and soil nutrient under different canopy species.

Canopy Species	Topography		Soil Nutrient Content		
	Slope (°)	Convexity (m)	Water (%)	Carbon (%)	Nitrogen (%)
*F. crenata*	26.74 ± 7.29	−0.13 ± 0.34	65.28 ± 1.69	16.46 ± 2.27	1.10 ± 0.15
*M. obovata*	30.49 ± 4.69	0.06 ± 0.33	68.29 ± 3.13	18.62 ± 3.54	1.24 ± 0.21

The detailed results of the Bayesian models are shown in Supplementary Table S2.

## Data Availability

The raw data supporting the conclusions of this article will be made available by the authors on request.

## Supplementary Materials

The following supporting information can be downloaded at https://www.mdpi.com/article/10.3390/plants13050719/s1: Figure S1: Experimental design; Table S1: The posterior distribution for each parameter of canopy openness; Table S2: The posterior distribution for each parameter of slope, convexity, soil water content, soil carbon content, and soil nitrogen content; Table S3: The posterior distribution for each parameter of leaf area, leaf dry weight, LMA, *P_nmax_*, and carbon and nitrogen per leaf and per leaf area; Table S4: The posterior distribution for each parameter of leaf area, leaf dry weight, LMA, *P_nmax_*, and carbon and nitrogen per leaf and per leaf area in different seasons; Table S5: The posterior distribution for each parameter of leaf area, leaf dry weight, LMA, *P_nmax_*, and carbon and nitrogen per leaf and per leaf area in phenological gaps; Table S6: The posterior distribution for each parameter of the interaction effects between canopy species and culm age on leaf characteristics in phenological gaps; Table S7: The posterior distribution for each parameter of the total number of leaves, number of dead leaves, and number of new leaves in different seasons; Table S8: The posterior distribution for each parameter of the interaction effects between canopy species and culm age on total number of leaves in phenological gaps; Table S9: The posterior distribution for each parameter of total length, average length, and average number of branches under in phenological gaps; Table S10: The posterior distribution for each parameter of above ground biomass; Table S11: The structure of models in Bayesian GLMM; Table S12: Differences in topography in the sub-plots used for each survey; Table S13: The posterior distribution of the slope and convexity parameters in the sub-plots used for each survey.
