# Supplementary material for: The Impact of Phenological Gaps on Leaf Characteristics and Foliage Dynamics of an Understory Dwarf Bamboo, Sasa kurilensis
Source: Plants (Basel). 2024 Mar 4;13(5):719. doi: 10.3390/plants13050719 (PMC10933764; doi:10.3390/plants13050719)
Supplement: Supplementary file 1 [file plants-13-00719-s001.zip › Figure S1.pdf]

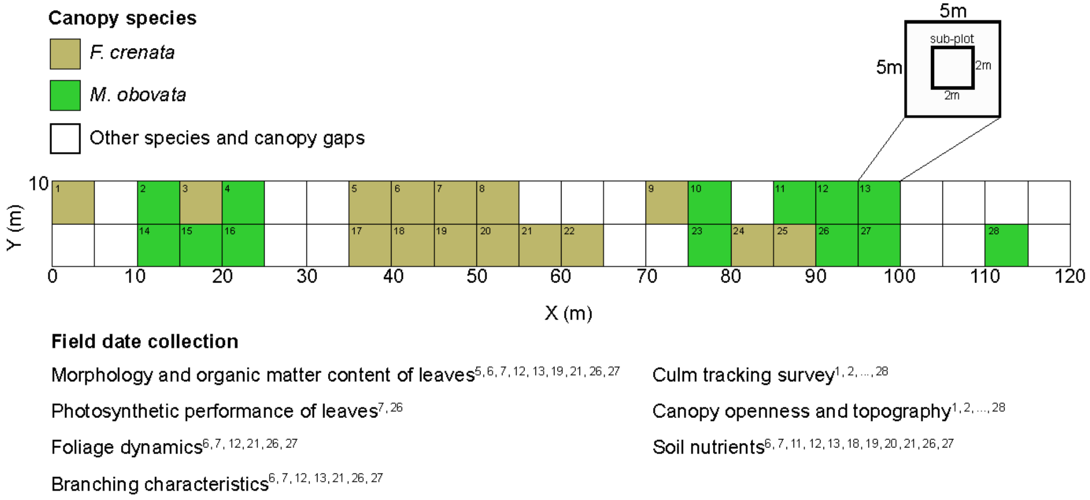

Figure S1. Canopy species distribution and field data collection sites across the study plot.
